# Author Correction: Evaluation of cognitive function in the Dog Aging Project: associations with baseline canine characteristics

**DOI:** 10.1038/s41598-023-34875-5

**Published:** 2023-05-30

**Authors:** Sarah Yarborough, Annette Fitzpatrick, Stephen M. Schwartz, Dog Aging Project Consortium, Joshua M. Akey, Brooke Benton, Elhanan Borenstein, Marta G. Castelhano, Amanda E. Coleman, Kate E. Creevy, Kyle Crowder, Matthew D. Dunbar, Virginia R. Fajt, Annette L. Fitzpatrick, Unity Jeffery, Erica C. Jonlin, Matt Kaeberlein, Elinor K. Karlsson, Kathleen F. Kerr, Jonathan M. Levine, Jing Ma, Robyn L. McClelland, Daniel E. L. Promislow, Audrey Ruple, Stephen M. Schwartz, Sandi Shrager, Noah Snyder-Mackler, M. Katherine Tolbert, Silvan R. Urfer, Benjamin S. Wilfond

**Affiliations:** 1 Department of Epidemiology, University of Washington, Seattle, WA USA; 2 Department of Family Medicine, University of Washington, Seattle, WA USA; 3 Epidemiology Program, Fred Hutchinson Cancer Research Center, Seattle, WA USA; 4 Lewis-Sigler Institute for Integrative Genomics, Princeton University, Princeton, NJ USA; 5 Department of Laboratory Medicine and Pathology, University of Washington School of Medicine, Seattle, WA USA; 6 Department of Clinical Microbiology and Immunology, Sackler Faculty of Medicine, Tel Aviv University, Tel Aviv, Israel; 7 Blavatnik School of Computer Science, Tel Aviv University, Tel Aviv, Israel; 8 Santa Fe Institute, Santa Fe, NM USA; 9 College of Veterinary Medicine, Cornell Veterinary Biobank, Cornell University, Ithaca, NY USA; 10 Department of Small Animal Medicine and Surgery, College of Veterinary Medicine, University of Georgia, Athens, GA USA; 11 Department of Small Animal Clinical Sciences, Texas A&M University College of Veterinary Medicine & Biomedical Sciences, College Station, TX USA; 12 Department of Sociology, University of Washington, Seattle, WA USA; 13 Center for Studies in Demography and Ecology, University of Washington, Seattle, WA USA; 14 Department of Veterinary Physiology and Pharmacology, Texas A&M University College of Veterinary Medicine & Biomedical Sciences, College Station, TX USA; 15 Department of Veterinary Pathobiology, Texas A&M University College of Veterinary Medicine & Biomedical Sciences, College Station, TX USA; 16 Institute for Stem Cell and Regenerative Medicine, University of Washington, Seattle, WA USA; 17 Bioinformatics and Integrative Biology, University of Massachusetts Chan Medical School, Worcester, MA USA; 18 Broad Institute of MIT and Harvard, Cambridge, MA USA; 19 Department of Biostatistics, University of Washington, Seattle, WA USA; 20 Division of Public Health Sciences, Fred Hutchinson Cancer Research Center, Seattle, WA USA; 21 Department of Biology, University of Washington, Seattle, WA USA; 22 Department of Population Health Sciences, Virginia-Maryland College of Veterinary Medicine, Virginia Tech, Blacksburg, VA USA; 23 Department of Biostatistics, Collaborative Health Studies Coordinating Center, University of Washington, Seattle, WA USA; 24 School of Life Sciences, Arizona State University, Tempe, AZ USA; 25 Center for Evolution and Medicine, Arizona State University, Tempe, AZ USA; 26 School for Human Evolution and Social Change, Arizona State University, Tempe, AZ USA; 27 Treuman Katz Center for Pediatric Bioethics, Seattle Children’s Research Institute, Seattle, WA USA; 28 Department of Pediatrics, Division of Bioethics and Palliative Care, University of Washington School of Medicine, Seattle, WA USA

Correction to: *Scientific Reports* https://doi.org/10.1038/s41598-022-15837-9, published online 25 August 2022

The original version of this Article contained errors in the Dog Aging Project Consortium list, where the following authors were omitted:

Joshua M. Akey, Brooke Benton, Elhanan Borenstein, Marta G. Castelhano, Amanda E. Coleman, Kyle Crowder, Matthew D. Dunbar, Virginia R. Fajt, Annette L. Fitzpatrick, Unity Jeffrey, Erica C. Jonlin, Elinor K. Karlsson, Kathleen F. Kerr, Jonathan M. Levine, Jing Ma, Robyn L. McClelland, Stephen M. Schwartz, Sandi Shrager, Noah Snyder-Mackler, M. Katherine Tolbert, Silvan R. Urfer, Benjamin S. Wilfond.

Consequently, the Supplementary Information file listing the Consortium members has been removed.

The original Article has been corrected.

